# Acupuncture May Reduce Insulin Doses in Overweight Insulin‐Treated Patients With Type 2 Diabetes Mellitus: A Crossover Randomized Controlled Pilot Trial

**DOI:** 10.1111/dom.70941

**Published:** 2026-06-03

**Authors:** Alessandro Milia, Giovanni Antonio Silverii, Lorenzo Cortoni, Matteo Monami, Vittorio Limatola, Edoardo Mannucci

**Affiliations:** ^1^ Internal Medicine, Careggi Hospital Florence Italy; ^2^ Diabetology, Careggi Hospital Florence Italy; ^3^ University of Florence Florence Italy; ^4^ Integrated Medicine Unit, Careggi Hospital Florence Italy

**Keywords:** clinical trial, insulin resistance, insulin therapy, type 2 diabetes

## 1 Background

Acupuncture is a Traditional Chinese Medicine (TCM) technique, based on the principle that needling specific points on the body surface (acupoints) may have therapeutic effects on some conditions, although its precise mechanism of action is unclear [1]. Acupuncture has been recognized as effective, and sometimes reimbursed [2], to treat different diseases [3]; it has also been proposed as a glucose‐lowering therapy in type 2 diabetes mellitus (T2DM) [4, 5], due to a possible insulin‐sensitizing effect as suggested by animal studies [6, 7]. In humans with and without T2DM, acupuncture was reported to reduce Body Mass Index (BMI) [8], fasting blood glucose and Homeostasis Model Assessment (HOMA) insulin resistance index [5, 9]; however, the reliability of the HOMA index in measuring insulin resistance in T2DM is controversial [10]. The aim of this study was to explore if acupuncture treatment may reduce insulin doses in insulin‐treated overweight people with T2DM, to collect further evidence on its hypothetical insulin sensitizing effect.

## 2 Methods

We performed a randomized controlled, single‐blinded trial with a crossover design, with blindness of the endpoint assessor. The protocol was registered in the Clinicaltrials.gov repository (NCT04076800 registration number), and summarized in Table S1,S3.

Adult overweight patients with T2DM, on insulin therapy for at least 6 months, with a dose greater than 20 IU daily, and a HbA1c < 69 mmol/mol, were eligible.

Patients underwent simple randomization process to acupuncture or sham in a 1:1 ratio (treatments are described in Table S2,S3). After treatment or sham completion, and a 1‐month washout period, patients on acupuncture or sham were switched to sham or acupuncture, respectively, in a cross‐sectional design (Figure S1).

No modifications were made to lifestyle or non‐insulin medications. Insulin dose was adjusted weekly by a diabetologist, blinded to the patient's current treatment, according to a prespecified protocol, based on patient‐reported glucose self‐monitoring (Table S4).

The primary objective was the superiority of acupuncture over sham in the reduction of daily insulin dose. The secondary endpoint was the difference in HbA1c from baseline (Table S2).

## 3 Results

The study was delayed soon after patients' enrolment due to the COVID‐19 pandemic; more recently, major advances in T2DM therapies significantly hampered our ability to enrol subjects in compliance with protocol: the approval of new non‐insulin agents which reduced the need for insulin in T2DM patients; the introduction of icodec insulin, which could not be included as concomitant medication due to different titration schemes; and the widespread increase in continuous glucose monitoring use, which limited the patients' willingness to perform traditional glucose tests. The reduced possibility of further enrolment prompted the decision to stop the trial.

At trial termination, 23 patients had signed the informed consent; of those, 3 had withdrawn during the first run‐in phase before randomization, whereas 5 had dropped out before completing the first treatment period (Figure S2). Because of the crossover nature of the trial, those patients were excluded from the analysis; an intention‐to‐treat analysis was not performed because no observation from the second treatment periods was available, including baseline values of endpoint variables. The remaining 15 patients completed both treatment phases, and they were included in the analysis. No patient was undergoing treatment at the time of trial termination.

The characteristics of the enrolled patients are summarized in Table S5. Eight patients were randomly assigned to acupuncture first, whereas seven started with sham. The mean daily total insulin dose at the beginning of the first and second treatment period was 52.5 ± 28.4 and 49.4 ± 28.0 IU, respectively (*p* = 0.28).

Acupuncture was associated with a mean reduction of total daily insulin of 5.3 IU, with a difference from sham of −13 [−8; −25]% (*p* = 0.039, Table 1).

**TABLE 1:** Difference in total insulin dose between acupuncture and sham.

	Acupuncture Mean ± SD	Sham Mean ± SD	Paired difference		*t*	*p*	Cohen *D*
			Mean ± SD	95% CI			Estimate (95% CI)
Total insulin dose difference (IU)	−5.27 ± 6.63	−1.67 ± 6.30	3.60 ± 6.5	(−0.01; 7.21)	2.14	0.050	0.55 (−0.001; 1.09)
Total insulin percentage difference (%)	−0.14 ± 0.20	−0.011 ± 0.15	0.13 ± 0.22	(0.08; 0.25)	2.28	0.039	0.50 (0.03; 1.13)
Short acting insulin dose difference (IU)	−2.00 ± 4.57	−0.83 ± 19.63	1.17 ± 19.48	(−11.21; 13.55)	0.21	0.839	0.060 (−0.51; 0.63)
Long acting insulin dose difference (IU)	−3.67 ± 4.7	1.93 ± 9.81	5.6 ± 11.90	(−0.99; 12.19)	1.82	0.090	0.47 (−0.07; 1.00)
HBA1c difference from baseline (mmol/mol)	−2.07 ± 4.59	+1.00 ± 5.69	3.07 ± 8.33	(−1.55; 7.68)	1.46	0,177	0.37 (−0.16; 0.89)
BMI difference from baseline (kg/m2)	−0.33 ± 0.51	−0.22 ± 0.49				0.107	

Abbreviations: CI = confidence interval; IU = international units; SD = standard deviations; *t* = paired samples student's *t*‐test.

**FIGURE 1:**
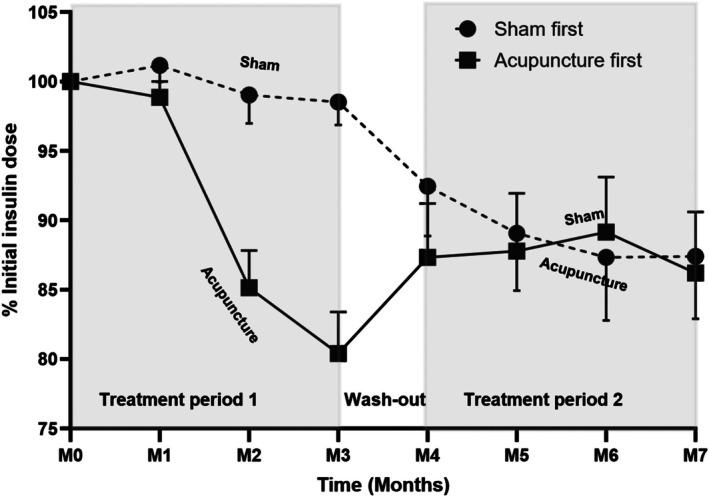
Variations in total insulin dose during the crossover trial: M0 = baseline (acupuncture first 54.25 ± 39.86 IU, sham first 50.71 ± 15.2 IU); M1 treatment month 1; M2; Treatment month 2; M3 = End of first treatment period (acupuncture first 48.38 ± 40.46, sham first); M4 = End of wash out, beginning of second treatment period (acupuncture first 50.00 ± 36.78 IU, sham first 48.71 ± 19.35 IU); M5; Treatment month 5; M6; Treatment month 6; M7 end of second treatment period (acupuncture first 46.88 ± 35.36 IU, sham first 46.00 ± 20.26 IU).

The post hoc calculated power (with *α* = 0.05) to detect as statistically significant the observed differences in absolute and percent reduction of insulin dose was 0.49 and 0.56, respectively. No significant difference in percent reduction of insulin doses with acupuncture vs. sham was observed between those who initiated with either the former or latter treatment (*p* = 0.48), ruling out any relevant carryover effect (Figure 1).

No significant difference between the two treatments was detected in mean daily doses of long‐ or short‐acting insulin, HbA1c and BMI (Table 1). Twelve (80%) and 11 (73.3%) patients were within the recommended HbA1c target as for local guidelines [11] after acupuncture or sham, respectively. No serious adverse events were reported (Table S6).

## 4 Conclusion

This trial suggests that acupuncture may reduce the insulin dose in T2DM patients, supporting the hypothesis of an insulin‐sensitizing effect. These results are in line with previous studies showing an improvement of insulin sensitivity with acupuncture [4, 5]; however, our study is the first to assess the effect of acupuncture in insulin‐treated patients with T2DM. The reduction of insulin doses indicated an effect of acupuncture on insulin sensitivity, overcoming the limits of HOMA index in patients with T2DM. The observed 5 IU mean reduction of daily insulin doses, which may be considered of moderate clinical significance, is similar to that observed by adding metformin to insulin therapy [12]. The absence of any effect on HbA1c was an expected consequence of trial design: the adjustment of insulin doses aimed at maintaining optimal glycaemic control did not allow differences of glucose levels between groups. Acupuncture was associated with a small weight loss, but differences between acupuncture and sham were not statistically significant and clinically trivial.

The present data do not demonstrate the efficacy of acupuncture in the treatment of T2DM, but support the hypothesis of a potential therapeutic use. Further RCTs, specifically designed for directly relevant clinical endpoints (e.g., HbA1c and hypoglycaemia) are needed for the assessment of efficacy and safety.

The present results should be considered only as hypothesis generating. The study design did not include specific measurements of insulin sensitivity (e.g., glucose infusion rate during euglycemic hyperinsulinemic clamp), of which daily insulin dose is only a proxy. In fact, a lower insulin dose could also have been theoretically determined by insulin undertreatment in the acupuncture arm; however, most patients were within the HbA1c targets in both treatment arms, making this hypothesis implausible. In addition, the termination of the study at about half of the planned enrolment reduces the reliability of results. Furthermore, long‐term effects treatment effects could not be assessed due to insufficient follow‐up length and cross‐over design; the cross‐over design also prevented an intention‐to‐treat analysis, given that none of the dropouts had started the second treatment period. Some patients may have dropped out because unable to follow the treatment schedule, or because they did not observe clinical benefit, thus potentially introducing a selection bias in favour of either acupuncture or sham.

In conclusion, a 3‐month acupuncture protocol may provide a greater reduction in insulin dose than a sham intervention in overweight people with T2DM in insulin therapy. Further studies on larger samples are needed to confirm this preliminary result.

## Author Contributions

A.M., and G.A.S., made the analysis plan, conducted the study, performed analyses, contributed to the discussion, and wrote the first draft of the manuscript. E.M. made the analysis plan, contributed to the discussion, and wrote the first draft of the manuscript. M.M., L.C. and V.L. contributed to study conduction, and reviewed and edited the manuscript. All the authors had full access to study data, approved the final version of the manuscript, and took responsibility for data integrity and analysis accuracy.

## Funding

The authors have nothing to report.

## Conflicts of Interest

G.A.S. has received a fee from Eli‐Lilly as a member of the advisory board; M.M. has received speaking fees from Astra Zeneca, Boehringer‐Ingelheim, Eli‐Lilly, Merck, Novo Nordisk and Sanofi. E.M. has received consultancy fees, speaking fees or research contributions from AstraZeneca, Bayer, Boehringer‐Ingelheim, Coresearch, Dexcom, Eli‐Lilly, Molteni, Novo Nordisk, Pikdare and Sanofi. A.M., L.C. and V.L. do not have any competing interests to disclose.

## Supporting information


**Table S1:** Trial procedures.
**Figure S1:**. Trial protocol flow chart.
**Table S2:** Protocol details.
**Table S3:** Acupuncture points.
**Table S4:**. Insulin therapy and concomitant treatments.
**Table S5:**. Baseline characteristics of the enrolled patients. SD = standard deviation; *n* = number *t*‐test student (included patients/drop out patients).
**Table S6:**. Adverse events table.
**Figure S2:**. Trial flow diagram.

## Data Availability

The data that support the findings of this study are available from the corresponding author upon reasonable request.

## References

[1] Q. Zhang , M. Zhou , M. Huo , et al., “Mechanisms of Acupuncture–Electroacupuncture on Inflammatory Pain,” Molecular Pain 19 (2023): 17448069231202882, 10.1177/17448069231202882. 37678839 PMC10515556

[2] M. T. Bordogna , “Regional Health Systems and Non‐Conventional Medicine: The Situation in Italy,” EPMA Journal 2 (2011): 411–423, 10.1007/s13167-011-0098-6. 23199178 PMC3405404

[3] H. L. Woo , H. R. Ji , Y. K. Pak , et al., “The Efficacy and Safety of Acupuncture in Women With Primary Dysmenorrhea: A Systematic Review and Meta‐Analysis,” Medicine (Baltimore) 97 (2018): e11007, 10.1097/MD.0000000000011007. 29879061 PMC5999465

[4] C. Chen , J. Liu , M. Sun , W. Liu , J. Han , and H. Wang , “Acupuncture for Type 2 Diabetes Mellitus: A Systematic Review and Meta‐Analysis of Randomized Controlled Trials,” Complementary Therapies in Clinical Practice 36 (2019): 100–112, 10.1016/j.ctcp.2019.04.004. 31383426

[5] S. Li , J. Chen , M. Liu , Y. P. Wang , X. Zhou , and X. Sun , “Effect and Safety of Acupuncture for Type 2 Diabetes Mellitus: A Systematic Review and Meta‐Analysis of 21 Randomised Controlled Trials,” Chinese Journal of Integrative Medicine 28 (2022): 463–471, 10.1007/s11655-021-3450-2. 34432205

[6] H.‐Y. Liao , M.‐F. Sun , J.‐G. Lin , S. L. Chang , and Y. C. Lee , “Electroacupuncture Plus Metformin Lowers Glucose Levels and Facilitates Insulin Sensitivity by Activating Mapk in Steroid‐Induced Insulin‐Resistant Rats,” Acupuncture in Medicine 33 (2015): 388–394, 10.1136/acupmed-2014-010724. 26025384

[7] X.‐Y. Huang , L. Zhang , J. Sun , N. G. Xu , and W. Yi , “Acupuncture Alters Expression of Insulin Signaling Related Molecules and Improves Insulin Resistance in OLETF Rats,” Evidence‐Based Complementary and Alternative Medicine 2016 (2016): 9651592, 10.1155/2016/9651592. 27738449 PMC5055976

[8] F. Ç. Tür , E. Aksay , T. Y. Kılıç , and Z. Temizyürek , “Therapeutic Effects of Acupuncture on Obesity and HbA1c,” European Journal of Integrative Medicine 7 (2015): 88–93, 10.1016/j.eujim.2014.12.002.

[9] T. Bai , X. Deng , J. Bi , L. Ni , Z. Li , and X. Zhuo , “The Effects of Acupuncture on Patients With Premature Ovarian Insufficiency and Polycystic Ovary Syndrome: An Umbrella Review of Systematic Reviews and Meta‐Analyses,” Frontiers in Medicine 11 (2024): 1471243, 10.3389/fmed.2024.1471243. 39655237 PMC11627218

[10] T. Pitea , G. Ionescu , E. Engelson , J. Albu , and D. Kotler , “Accuracy of HOMA‐IR in Clinical Practice: 342,” American Journal of Gastroenterology 104 (2009): S129, 10.14309/00000434-200910003-00342.

[11] E. Mannucci , R. Candido , L. D. Monache , et al., “Italian Guidelines for the Treatment of Type 2 Diabetes,” Acta Diabetologica 59 (2022): 579–622, 10.1007/s00592-022-01857-4. 35288805 PMC8995274

[12] B. Hemmingsen , L. L. Christensen , J. Wetterslev , et al., “Comparison of Metformin and Insulin Versus Insulin Alone for Type 2 Diabetes: Systematic Review of Randomised Clinical Trials With Meta‐Analyses and Trial Sequential Analyses,” BMJ 344 (2012): e1771, 10.1136/bmj.e1771. 22517929

