# Supplementary material for: Acupuncture May Reduce Insulin Doses in Overweight Insulin‐Treated Patients With Type 2 Diabetes Mellitus: A Crossover Randomized Controlled Pilot Trial
Source: Diabetes Obes Metab. 2026 Jun 3;28(8):7622–5. doi: 10.1111/dom.70941 (PMC13341373; doi:10.1111/dom.70941)
Supplement: Supplementary file 1 — Table S1: Trial procedures. Figure S1:. Trial protocol flow chart. Table S2: Protocol details. Table S3: Acupuncture points. Table S4:. Insulin therapy and concomitant treatments. Table S5:. Baseline characteristics of the enrolled patients. SD = standard deviation; n = number t‐test student (included patients/drop out patients). Table S6:. Adverse events table. Figure S2:. Trial flow diagram. [file DOM-28-7622-s001.docx]

| **Week** | **-4** | | **-2** | | **0** | | **1** | | **2** | | **3** | | **4** | | **5** | | **6** | | **7** | | **8** | | **9** | | **10** | | **11** | | **12** | | **13** | | **14** | | **15** | | **16** | | **17** | | **18** | | **19** | | **20** | | **21** | | **22** | | **23** | | **24** | | **25** | | **26** | | **27** | |  |
| --- | --- | --- | --- | --- | --- | --- | --- | --- | --- | --- | --- | --- | --- | --- | --- | --- | --- | --- | --- | --- | --- | --- | --- | --- | --- | --- | --- | --- | --- | --- | --- | --- | --- | --- | --- | --- | --- | --- | --- | --- | --- | --- | --- | --- | --- | --- | --- | --- | --- | --- | --- | --- | --- | --- | --- | --- | --- | --- | --- | --- | --- |
| **Visit** | **1** | **2** | **3** | **4** | **5** | **6** | **7** | **8** | **9** | **10** | **11** | **12** | **13** | **14** | **15** | **16** | **17** | **18** | **19** | **20** | **21** | **22** | **23** | **24** | **25** | **26** | **27** | **28** | **29** | 30 | **31** | **32** | **33** | **34** | **35** | **36** | **37** | **38** | **39** | **40** | **41** | **42** | **43** | **44** | **45** | **46** | **47** | **48** | **49** | **50** | **51** | **52** | **53** | **54** | **55** | **56** | **57** | **58** | **59** | **60** |  |
| Inclusion criteria | **x** |  |  |  |  |  |  |  |  |  |  |  |  |  |  |  |  |  |  |  |  |  |  |  |  |  |  |  |  |  |  |  |  |  |  |  |  |  |  |  |  |  |  |  |  |  |  |  |  |  |  |  |  |  |  |  |  |  |  |  |  |
| Consent | **x** |  |  |  |  |  |  |  |  |  |  |  |  |  |  |  |  |  |  |  |  |  |  |  |  |  |  |  |  |  |  |  |  |  |  |  |  |  |  |  |  |  |  |  |  |  |  |  |  |  |  |  |  |  |  |  |  |  |  |  |  |
| Medical History | **x** |  |  |  |  |  |  |  |  |  |  |  |  |  |  |  |  |  |  |  |  |  |  |  |  |  |  |  |  |  |  |  |  |  |  |  |  |  |  |  |  |  |  |  |  |  |  |  |  |  |  |  |  |  |  |  |  |  |  |  |  |
| Body Exam | **x** |  |  |  |  |  |  |  |  |  |  |  |  |  |  |  |  |  |  |  |  |  |  |  |  |  |  |  |  |  |  |  |  |  |  |  |  |  |  |  |  |  |  |  |  |  |  |  |  |  |  |  |  |  |  |  |  |  |  |  |  |
| Body weight |  |  | **x** |  |  |  |  |  | **x** |  |  |  |  |  |  |  | **x** |  |  |  |  |  |  |  | **x** |  |  |  |  |  |  |  |  |  | **x** |  |  |  |  |  | **x** |  |  |  |  |  |  |  | **x** |  |  |  |  |  |  |  | **x** |  |  |  |  |
| Randomization | **x** |  |  |  |  |  |  |  |  |  |  |  |  |  |  |  |  |  |  |  |  |  |  |  |  |  |  |  |  |  |  |  |  |  |  |  |  |  |  |  |  |  |  |  |  |  |  |  |  |  |  |  |  |  |  |  |  |  |  |  |  |
| Treatment |  |  | **x** | **x** | **x** | **x** | **x** | **x** | **x** | **x** | **x** | **x** | **x** | **x** | **x** | **x** | **x** | **x** | **x** | **x** | **x** | **x** | **x** | **x** | **x** | **x** |  |  |  |  |  |  |  |  | **x** | **x** | **x** | **x** | **x** | **x** | **x** | **x** | **x** | **x** | **x** | **x** | **x** | **x** | **x** | **x** | **x** | **x** | **x** | **x** | **x** | **x** | **x** | **x** |  |  |  |
| AE |  |  | **x** | **x** | **x** | **x** | **x** | **x** | **x** | **x** | **x** | **x** | **x** | **x** | **x** | **x** | **x** | **x** | **x** | **x** | **x** | **x** | **x** | **x** | **x** | **x** | **x** |  |  |  |  |  |  |  | **x** | **x** | **x** | **x** | **x** | **x** | **x** | **x** | **x** | **x** | **x** | **x** | **x** | **x** | **x** | **x** | **x** | **x** | **x** | **x** | **x** | **x** | **x** | **x** | **x** | **x** |  |
| Hba1c |  |  | **x** |  |  |  |  |  |  |  |  |  |  |  |  |  |  |  |  |  |  |  |  |  |  |  | **x** |  |  |  |  |  |  |  | **x** |  |  |  |  |  |  |  |  |  |  |  |  |  |  |  |  |  |  |  |  |  |  |  | **x** |  |  |
| Insulin titration | **x** | **x** | **x** |  | **x** |  | **x** |  | **x** |  | **x** |  | **x** |  | **x** |  | **x** |  | **x** |  | **x** |  | **x** |  | **x** |  | **x** |  |  |  |  |  |  |  | **x** |  | **x** |  | **x** |  | **x** |  | **x** |  | **x** |  | **x** |  | **x** |  | **x** |  | **x** |  | **x** |  | **x** |  | **x** |  |  |
| Wash out |  |  |  |  |  |  |  |  |  |  |  |  |  |  |  |  |  |  |  |  |  |  |  |  |  |  | **x** | **x** | **x** | **x** | **x** | **x** | **x** | **x** |  |  |  |  |  |  |  |  |  |  |  |  |  |  |  |  |  |  |  |  |  |  |  |  |  |  |  |
| Run In | x |  |  |  |  |  |  |  |  |  |  |  |  |  |  |  |  |  |  |  |  |  |  |  |  |  |  |  |  |  |  |  |  |  |  |  |  |  |  |  |  |  |  |  |  |  |  |  |  |  |  |  |  |  |  |  |  |  |  |  |  |

**Table S1:** trial procedures

**Figure S1:** trial protocol flow chart

**Exclusion criteria:** The main exclusion criteria were: inadequately treated Axis I mental disorders; current pregnancy or breastfeeding, or pregnancy planning; Corticosteroid treatment; Severe heart failure, renal ,liver or respiratory failure (oxygen therapy).

**Statistics:** The variables of interest were: the difference between mean total insulin dose, short-acting insulin dose and long-acting insulin dose variations between baseline and the end of acupuncture of sham treatments; the difference between percentage of median total insulin dose reduction from baseline, and the difference between HbA1c at the end of the treatments. All these variables were assessed using paired Student t-tests. To rule-out a carry-over effect, the mean difference between the effects on the principal endpoint between acupucture and sham was calculated separately in patients starting with acupuncture or sham, and the groups with the two treatment sequences were then compared using an unpaired samples t-test.
Power calculation: given the 2.3±8.73 IU observed 6-month insulin dose variance in a sample of 64 consecutive outpatients who had attended two visits in our hospital in 2017 (whose mean insulin basal dose was 52.3 UI), we hypothesized a 42 patients sample as sufficient to detect a 15% insulin dose reduction from baseline as significant with a p=0.05, a 80% power, and a 10% dropout.
Randomization list and analyses were performed using IBM SPSS Statistics™, version 29.0.2.0.

**ACUPUNCTURE AND SHAM DETAILS**

**1) Rationale for Acupuncture**

**1a) Type of Acupuncture Used**
The selection of acupuncture points and stimulation methods is based on the principles of Traditional Chinese Medicine (TCM), specifically the Zang-Fu (Organ-Viscera) theory in relation to the *Xiao-Ke* syndrome (also known as *Tang Niao Bing*), the traditional counterpart of Type 2 Diabetes Mellitus (T2DM) in the modern interpretation of TCM (15).

**1b) Treatment Details**
In TCM, Diabetes Mellitus is referred to as *Xiao Ke* (“consumptive thirst”) or *Tang Niao Bing* (“sweet urine disease”). Clinically, *Xiao Ke* syndrome can be subdivided into three categories: *Shang Xiao* (Upper Diabetes), *Zhong Xiao* (Middle Diabetes), and *Xia Xiao* (Lower Diabetes), each respectively characterized by polydipsia, polyphagia, and polyuria.
From a Western perspective, *Shang Xiao* and *Xia Xiao* correspond to decompensated stages of diabetes. As the study population will consist of patients with good or acceptable glycometabolic control, the selection of acupoints primarily targets *Zhong Xiao* syndrome, which manifests predominantly as polyphagia—more commonly observed in T2DM patients.

Point selection is also supported by existing literature regarding the effects of acupuncture on glucose metabolism in healthy, obese, and T2DM subjects (6–11). The acupoints selected for the basic treatment are: SP6, ST36, KI3, CV4, CV12, TE5, LI4, LI11, YinTang, ST28, BL23, BL22, BL20, BL13.

**2) Technical Details of Treatment**

**2a) Number of Acupoints per Subject per Session**
Subjects in the true acupuncture group will be treated at 14 basic acupoints (11 of which are bilateral). At the end of each session, 3 auricular points will be selected according to the criteria described above.

**2b) Names and Localization of Acupoints**
Acupoint localization will be determined based on anatomical landmarks combined with palpatory assessment of the area to identify the most compliant, tender, or pressure-sensitive point.

**2c) Depth of Needle Insertion**
For abdominal acupoints, sterile disposable needles (0.25 × 40 mm; 0.25 × 60 mm) will be inserted after skin disinfection, to variable depths depending on subcutaneous fat thickness, up to the resistance of the superficial muscle fascia (maximum 6 cm), with perpendicular insertion to the skin, except for CV12, which will be angled approximately 30° toward the umbilicus.

For dorsal acupoints, sterile disposable needles (0.25 × 30 mm) will be inserted after skin disinfection to a depth corresponding to 1 *cun* (1/6 of the interscapular distance), obliquely directed toward the gluteal region.

For limb acupoints, sterile disposable needles (0.25 × 25 mm) will be inserted after skin disinfection to a maximum depth of 1 *cun* (1/12 of the distance between the ulnar wrist margin and the ulnar trochlea at the arm, and 1/15 of the distance between the lateral malleolus and lateral femoral condyle in the leg), perpendicularly (SP6, KI3, ST36) or obliquely directed upward (TE5).

For cranial acupoints, sterile disposable needles (0.25 × 25 mm) will be inserted tangentially toward the nasal root (YinTang) at a depth of approximately 0.5 *cun* (corresponding to 1/18 of the distance between the two mastoid processes and 1/6 of the distance between the hairline and eyebrow ridge).

At the auricular level, sterile disposable semi-permanent Seirin© needles (0.2 × 0.3 mm) will be applied to the three selected points, identified by tenderness upon pressure.

Auricular acupuncture, which was also included in the original protocol, was not applied in any of the patients that completed the trial procedures, because of difficulties in making the sham auricular procedure indistinguishable from acupuncture from the patient’s perspective. During the 3-month treatment period, patients underwent 2 sessions every week, lasting 25 minutes each. At the beginning of each treatment, the acupuncturist performed needle manipulation, thus, eliciting the *de qi* sensation[1]. Based on points disposition, the needle might be manipulated to tonify or reduce, according to TCM principles (see Table S2 in the appendix for details).

**2d) Desired Response**
Before needle insertion, the active point will be identified through light palpation, seeking greater compliance, tenderness, or sensory changes (numbness, electric sensation), either perceived by the practitioner or reported by the patient.
After insertion, the practitioner will seek the *De Qi* response (“arrival of Qi”), perceived by the patient as numbness, soreness, electric shock, or local/distant warmth, and by the acupuncturist as increased needle stability during manipulation.

**2e) Needle Manipulation**
Needle manipulation will follow TCM techniques of tonification (SP6, KI3, ST36, CV4, CV12, BL23, BL22, BL20, BL13), dispersion (LI11), and harmonization (YinTang, TE5, LI4).

Tonification: Needles will be slightly angled along the meridian direction and rotated slowly with small amplitude toward the body’s midline (clockwise for CV4 and CV12), combined with lifting-thrusting movements faster in the thrusting phase.

Dispersion: Needles will be slightly angled opposite the meridian flow, rotated rapidly with large amplitude toward the midline, and manipulated with lifting-thrusting movements faster in the lifting phase.

Harmonization: Needles will be inserted perpendicularly and rotated slowly in alternating directions, accompanied by equally paced lifting-thrusting movements.

Patients will be instructed to manually stimulate the auricular semi-permanent needles daily between sessions using light rotations and pressure.

**2f) Needle Retention Time**
Needles will be retained for 20 minutes on the anterior body and 20 minutes on the posterior body, for a total treatment duration of 40 minutes.

**2g) Needle Diameter**
Sterile disposable needles with a diameter of 0.25 mm and length ranging from 25 to 60 mm will be used (40–60 mm for abdominal areas or in patients with thicker subcutaneous fat).
For auricular acupuncture, sterile disposable semi-permanent needles (0.2 × 0.3 mm) will be used.

**3) Treatment Regimen**

**3a) Number of Sessions**Two sessions per week will be performed, spaced at least 48 hours apart, over 12 consecutive weeks, for a total of 24 sessions.

**4) Additional Treatment Information**

**4a) Adjunct Techniques**No adjunctive techniques (such as moxibustion, cupping, bloodletting, herbal administration, or therapeutic exercises such as Tai Chi or Qi Gong) will be used.

**4b) Patient Instructions**All patients will provide written informed consent for acupuncture and will be informed of potential side effects and complications (vasovagal reaction with possible pre-syncope or syncope, minor bleeding or hematoma at insertion site, pneumothorax), including their frequency and severity.
Patients will be instructed to massage the auricular semi-permanent needles daily.
All treatments will be performed at the *Careggi University Hospital* in Florence, within the outpatient clinics of the Division of Diabetology directed by Prof. Edoardo Mannucci.

**5) Acupuncturist’s Experience**

All acupuncture sessions will be performed by a single acupuncturist registered with the *Federazione Italiana Società di Agopuntura* (F.I.S.A.), holding a diploma from the *Associazione dei Medici Agopuntori Bolognesi* (A.M.A.B.), accredited by the Emilia-Romagna Region (Determination No. 5050, 05/04/2017). The qualification is nationally recognized and equivalent to a “university master’s degree” in Acupuncture (see annexes).

**6) Control Group**

**6a) Rationale for the Control Group**The control group will receive sham acupuncture. This control aims to minimize procedural differences between groups, acknowledging that sham acupuncture is not entirely inert (placebo) but only minimally effective.To reduce confounding therapeutic effects, the sham points will be located in dermatomes distinct from the true acupoints, inserted superficially (1–2 mm) within the skin layer, outside the pathways of the 12 main TCM meridians.
No point palpation will be performed before insertion, and needles will be inserted using guide tubes to minimize physical contact between practitioner and patient.
Sterile disposable needles will be used.
Sham-treated patients will undergo 20 minutes in the supine position and 20 minutes in the prone position per session, for a total of 40 minutes, twice weekly over 12 weeks (24 sessions in total).
by selecting the points aimed at treating Xiao-Ke or Tang Niao Bing syndrome, *Zhong Xiao* or *Pi Dan* subdivisions [2]. Those symptoms identify T2DM modern patients in the TCM view. Point selected for treatment were: KI3; SP6; ST36; CV4; CV12; TE5; LI4; LI11; ST28; BL23; BL22; BL20; BL13[3]. These acupuncture points were selected based on their therapeutic actions, according to the principles of Traditional Chinese Medicine, in addressing metabolic and digestive dysfunctions (SP6; ST36; CV12; LI4; ST28; BL20; TE5; BL22), as well as the presence of internal heat and kidney deficiency (TCM’s pattern of disease) (KI3; CV4; BL23; LI11; BL13), which are commonly associated with type 2 diabetes mellitus in the patterns identified by Traditional Chinese Medicine (greater details on the appendix).

**Details of Sham treatment**

In the Sham group, the treatment consists of inserting three sterile, single-use needles using a guide tube at the following body regions: the posterolateral surface of the thigh, at the S1 dermatome level, approximately halfway between the main BL meridian (running slightly medial to the midline) and the GB channel (running along the lateral portion of the thigh), at mid-thigh level; the posterolateral surface of the calf, at the S1 dermatome level, approximately halfway between the main BL meridian (running slightly medial to the midline) and the GB channel (running along the fibula), at the proximal third of the leg; and the upper third of the arm, midway between the LI and TE channels.In each of these regions, three needles are inserted to a depth of approximately 1-3 mm, arranged to form an equilateral triangle with 1 cm sides. After insertion, the needles are not manipulated and are left in place for a total duration of 20 minutes.

**Medical Devices Used:** In the present study, acupuncture needles (category code **A019002**), classified as **Class IIa medical devices** (Annex IX, Legislative Decree 46/97), will be used.The devices are manufactured by **Asia-Med GMBH & CO. KG**, model **TeWa5CBAR** (device registration number **1680115**), and by **Seirin Corporation**, model **Pyonex press needles** (device registration number **181974**).

**Instructions for Use:** The procedures for the use of acupuncture needles are described in detail in **Section 2 of the STRICTA criteria**. According to **EU Regulation 2017/745**, *Instructions for Use (IFU)* are not required for Class IIa medical devices, such as acupuncture needles, when they can be used safely without such documentation.The use of acupuncture needles requires proficiency in manual technique and a thorough understanding of the underlying rationale—in this case, the principles of Traditional Chinese Medicine (see Section 1 of the STRICTA criteria).

**Table S2** Protocol details

|  | **Acupuncture** | **Sham** |
| --- | --- | --- |
| **Points** | SP6; ST36; KI3; CV4; CV12; TE5; LI4; LI11; YinTang; ST28; BL23; BL22; BL20; BL13. | Points outside main meridians, on arms and legs; see table S2 for exact location |
| **Needeling manipulation** | Armonization, tonification or dispersion based on the point (see 2e paragraph) reaching the De Qi sensation | No manipulation |
| **Depth of needle insertion** | Depending on the point (See 2c for details) | Superficial (1-3 mm) |

**Table S3** Acupuncture points

**Table S4. Insulin Therapy and Concomitant Treatments**

At the time of randomization, all concomitant pharmacological treatments and any subsequent modifications during the study will be recorded. Long-acting insulin was modified according to fasting blood glucose, and short-acting insulin was modified according to post-prandial blood glucose, with a target of a 0-60 mg/dl increase from pre-prandial value. If, in the opinion of the investigator, a patient fails to maintain adequate glycemic control, **insulin therapy adjustments** may be implemented according to the following algorithm.

| **Mean fasting blood glucose (mg/dl) (3 previous days)** | **Long-acting Insulin dose variation from previous (IU)** |
| --- | --- |
| 91–120 | None |
| 121 – 146 | +2 |
| 147–164 | +4 |
| 165-182 | +6 |
| >182 | +8 |
| **Lower blood glucose value(mg/dl)** | **Long-acting Insulin dose variation (IU)** |
| < 76 | -4 (or 10% if dose >45 U) |
| 76 – 85 | -2 (or 5% if dose >45 U) |
| 85- 91 | -1 |
| 91-128 | None |
| >128 | +1 |
| **Blood Glucose before each meal (mg/dl)** | **short-acting insulin dose variation (U)** |
| < 91 | -1 |
| 91-128 | None |
| >128 | +1 |
| **Blood glucose 2 h after each meal (mg/dl)** | **short-acting insulin dose variation (U)** |
| -20 mg/dl from pre-prandial | 10-15% reduction |
| -20 mg/dl/+60 mg/dl from pre-prandial | None |
| +60 mg/dl increase from pre-prandial | 10-15% increase |

**Table S5:** Baseline Characteristics of the enrolled patients. SD= Standard Deviation; n= number

**T-test student (included patients /drop out patients)**

| **Baseline Characteristics Mean± SD (minimum, maximum)** | **Included patients (n=15)** | **Not included patients (n=8)** | **P** |
| --- | --- | --- | --- |
| Age (n=15) | 65.73±7.03 (53, 78) | 64.5 ± 9.23 (59,76) | 0.77 |
| HbA1c (n=15) | 54.87±6.27 (44,66) | 57.7 ± 6.01 (48,69) | 0.21 |
| Weight (n=15) | 101.20±18.13 (74, 145) | 94.7 ± 9.92 (81,106) | 0.12 |
| BMI (n=15) | 34.21±4.83 (25.34, 42.83) | 30.0 ± 4.26 (25.5, 38) | 0.07 |
| Long Acting insulin dose (n=15) | 30.07±12.70 (12, 52) | 33 ± 20.57 (10,79) | 0.68 |
| Short Acting insulin dose | 34.00±23.32 (0, 68) (n=9) | 41.6 ± 36.2 (8,124) (n=6) | 0.63 |
| Total Insulin Dose (n=15) | 52.47±28.45 (12, 120) | 74.6 ± 53.9 | 0.29 |
| **Non insulin-medications, n (%)** |  |  |  |
| Metformin | 13 (86,7) | 5 (62.5 %) | 0.18 |
| SGLT2-inhibitors | 9 (60%) | 6 (75 %) | 0.47 |
| GLP1-Receptor agonists | 10 (66.6%) | 2 (25 %) | 0.056 |

| **Adverse events** | | |
| --- | --- | --- |
|  | **During Acupuncture period** | **During Sham period** |
| Localized self-limiting cutaneous irritation | 2/15 | 1/15 |
| Minor self-limiting bleeding | 1/15 | 0/15 |

**Table S6:** Adverse events table


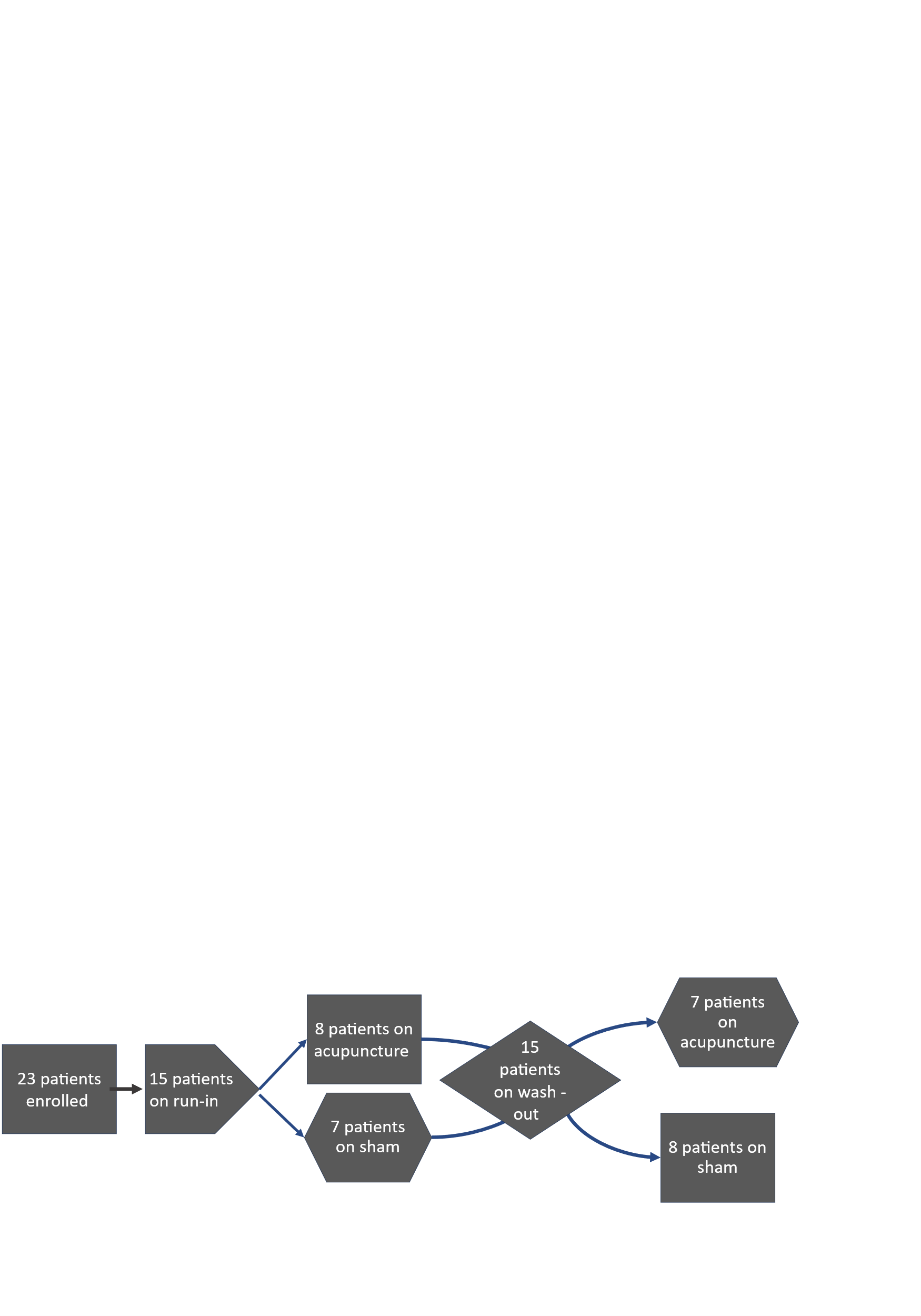


**Figure S2:** Trial Flow Diagram

**REFERENCES**

1. Yang X-Y, Shi G-X, Li Q-Q, Zhang Z-H, Xu Q, Liu C-Z (2013) Characterization of Deqi Sensation and Acupuncture Effect. Evid Based Complement Alternat Med 2013:1–7. https://doi.org/10.1155/2013/319734

2. Lian F, Ni Q, Shen Y, et al (2020) International traditional Chinese medicine guideline for diagnostic and treatment principles of diabetes. Ann Palliat Med 9(4):2237–2250. https://doi.org/10.21037/apm-19-271

3. Standard acupuncture nomenclature : a brief explanation of 361 classical acupuncture point names and their multilingual comparative list. World Health Organization. Regional Office for the Western Pacific
